# Satisfactory short-term outcomes of totally laparoscopic ileostomy reversal compared to open surgery in colorectal cancer patients

**DOI:** 10.3389/fsurg.2022.1076874

**Published:** 2023-01-06

**Authors:** Zheng Xu, Shou Luo, Hao Su, Jianwe Liang, Qian Liu, Xishan Wang, Weisen Jin, Haitao Zhou

**Affiliations:** ^1^ Department of Colorectal Surgery, National Cancer Center/National Clinical Research Center for Cancer/Cancer Hospital, Chinese Academy of Medical Sciences and Peking Union Medical College, Beijing, China; ^2^ Department of Gastrointestinal Surgery, Key Laboratory of Carcinogenesis and Translational Research (Ministry of Education/Beijing), Peking University Cancer Hospital & Institute, Beijing, China; ^3^ Department of Anorectal Diseases, General Hospital of Chinese Armed Police Forces, Beijing, China

**Keywords:** colorectal cancer, ileostomy reversal, intracorporeal anastomosis, surgical oncology, total laparoscopic surgery

## Abstract

**Background:**

Recently, totally laparoscopic (TLAP) surgery has suggested its potential on ileostomy reversal. This study aimed to compare the short-term outcomes between TLAP and traditional open ileostomy reversal.

**Patients and methods:**

From September 2016 to September 2021, 107 eligible patients underwent TLAP (*n* = 48) or open (*n* = 59) loop ileostomy reversal were retrospectively enrolled. Surgical parameters, postoperative recovery and complications were identified and compared between TLAP technique vs. open surgery.

**Results:**

The operation time and estimated blood loss showed no obvious difference between TLAP and open group. However, TLAP reversal significantly decreased the incision length (4.5cm vs. 6cm, *P* < 0.001). Furthermore, patients underwent TLAP surgery showed quicker first ground activities (1 day vs. 2 days, *P* < 0.001), faster first flatus passage (2 days vs. 3 days, *P* = 0.004) and shorter postoperative stay (5 days vs. 7 days, *P* = 0.007). More importantly, postoperative complications were significantly reduced after TLAP reversal (3 cases vs. 10 cases, *P* = 0.026). Further logistic regression analyses also indicated the TLAP technique was associated with lower incidence of complications (OR=3.316, CI, 1.118–9.835; *P* = 0.031).

**Conclusions:**

TLAP surgery is competitive in promoting postoperative recovery as well as reducing complications compared to the traditional open ileostomy reversal.

## Introduction

In order to protect the downstream anastomoses and avoid anastomotic leakage, loop ileostomy is frequently used in colorectal cancer surgery (1). However, loop ileostomy often leads to a plethora of postoperative complications, decline of quality of life and some psychological problems (2, 3). To reduce complications and increase quality of life, some researchers have applied a skin bridge technique to loop ileostomy (4). Apart from that, a timely reversal is also paramount (5). Nevertheless, ileostomy reversal has an estimated 17.3% morbidity (such as small bowel obstruction, wound infection and incisional hernia et al.) and 0.4% mortality (6). These postoperative complications were commonly reported even for senior surgeons. Given to these facts above, a safe and feasible approach of ileostomy reversal is in great demand.

As minimally invasive techniques evolved, laparoscope has been increasingly applied to colorectal operations. For instance, some researchers have shown the laparoscopic reversal was associated with rapid postoperative recovery, reduced morbidity and lower cost compared to open approach (7, 8). Satisfactory outcomes were also reported in patients with Crohn's disease and obesity (9, 10). When focusing on anastomotic technique itself, laparoscopic reversal made the intracorporeal digestive tract reconstruction possible. In recent years, some initial attempts have been made on laparoscopic-assisted intracorporeal anastomosis (11, 12). However, the feasibility of totally laparoscopic (TLAP) ileostomy reversal on colorectal cancer patients was not fully elaborated.

So far, TLAP right hemicolectomy has presented quicker recovery of bowel function than laparoscopic-assisted surgery with extracorporeal ileocolic anastomosis (13). Our previous report also revealed the strength of TLAP in obese patients (10). Based on these foundations above, this study aimed to compare the short-term outcomes between TLAP and open ileostomy reversal in Chinese colorectal cancer patients.

## Materials and methods

### Patients

From September 2016 to September 2021, consecutive patients underwent loop ileostomy reversal were enrolled in this retrospective study. All patients had a previous history of laparoscopic colorectal operation and received either TLAP reversal (48 cases) or open surgery (59 cases) by a single senior surgeon at the National Cancer Center/National Clinical Research Center for Cancer/Cancer Hospital, Chinese Academy of Medical Sciences and Peking Union Medical College. The senior operator was trained as an oncology surgeon for 20 years with extensive open (over 500 procedures since 2010) and laparoscopic colorectal surgery experiences (more than 100 procedures/year since 2015). Potential benefits and defects were explained to all patients concretely and the informed contents were signed before every reversal operation. Certainly, this study was approved by the Ethical Committee of the Cancer Hospital (Institute), Chinese Academy of Medical Sciences, Beijing, People's Republic of China and in accordance with the ethical standards of the Declaration of Helsinki.

Of note, any patients suited for the classic open reversal were regarded as potential candidates for TLAP surgery. As a result, eligible patients could freely select the operation approach based on their preference when TLAP technique was introduced to our group in the latter half of 2018. For all patients, the reversal of ileostomy should be performed at least 3 months after primary colorectal cancer surgery or 8 weeks after postoperative chemotherapy/radiotherapy in patients aged above 18. Moreover, a colonoscope examination should also be performed before the reversal to ensure a good healing of the anastomotic stoma. In addition, an enhanced computed tomography of thoracic, abdominal and pelvic cavity was also routinely used to exclude tumor recurrence or metastasis. As for the exclusion criteria, operations featured ancillary procedures such as parastomal hernia/abdominal wall repair, anastomotic reconstruction or additional bowel resection were excluded for further data analyses. Patients with physical disability, cognition impairment or mental disturbance that would prohibit the understanding of informed consent and interfere with regular follow-up were also considered as exclusion criteria based on previous literatures (14, 15).

### Surgical procedure

**Figure 1:**
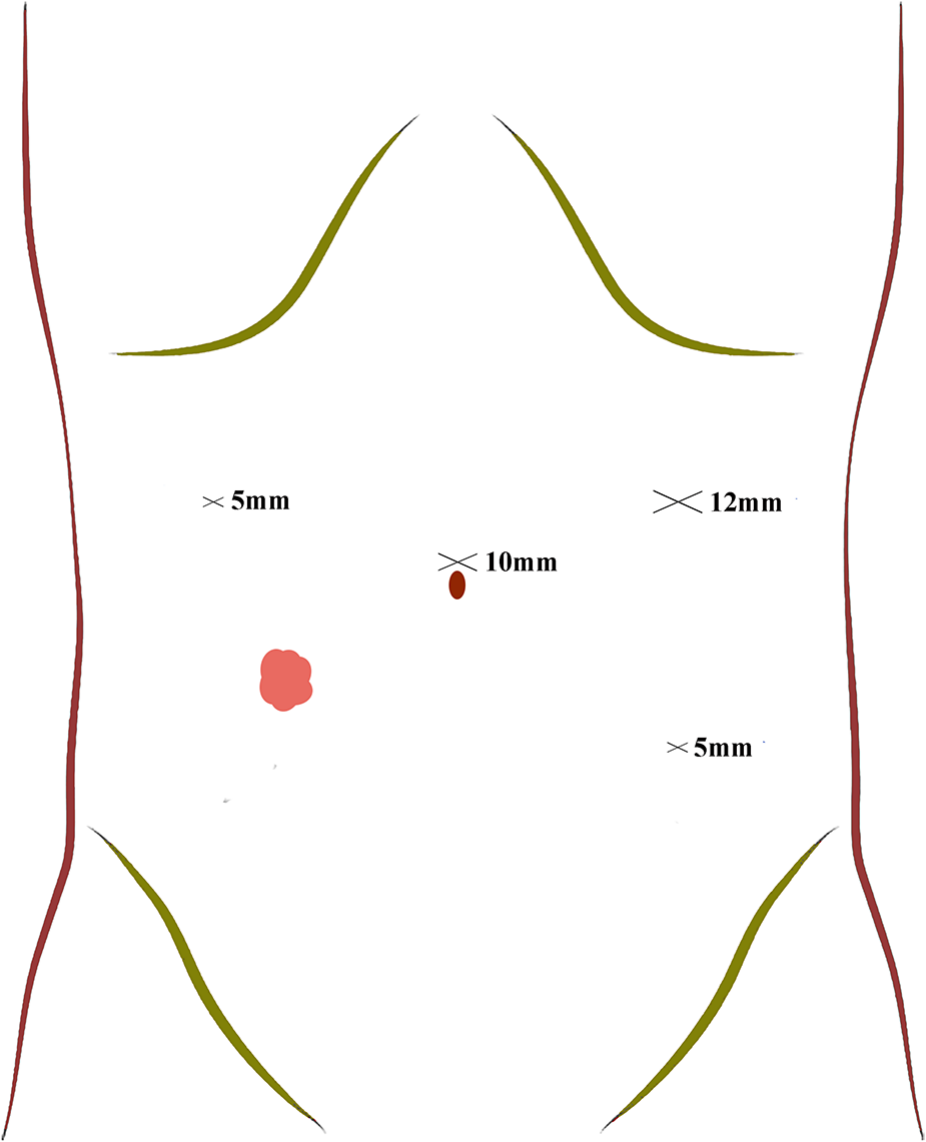
Trocar position and the size of trocars.

After general anesthesia, patients in TLAP group were placed in a supine lithotomy position. Here, a four-port procedure was routinely applied to place trocars (Figure 1). Firstly, a 10 mm supraumbilical port was used as the observation port. Then, a 12 mm supraumbilical port located in the left anterior axillary line was used as the principle operating port and a 5 mm port in the left lower quadrant was the auxiliary operating port. Another 5 mm ports located in the right anterior axillary line 10 cm superior the stoma was used as secondary operating port for assistants. After trocars placement, an ultrasonic scalpel was used to separate the adhesions around the stoma, identify the loop ileostomy and dissect the mesenteries. The transection of proximal and distal ileum was subsequently performed using a 60-mm endoscopic linear stapler (Johnson ECR60B).

As concerned the digestive tract reconstruction, the intracorporeal delta-shaped anastomosis was applied. In brief, two ends of the proximal and distal intestines were folded. After that, two 1 cm incisions located at the anti-mesenteric side of the intestine ends were made respectively and sterilized for further anastomosis. Subsequently, a side-to-side anastomosis was performed and the lumens were imbedded with a 60-mm endoscopic linear cutter stapler (Johnson ECR60B), followed by the connection of intestinal walls with no mesentery. Next, the common opening of the two intestines were closed by another endoscopic linear cutter stapler and the mesenteric defect was stitched by absorbable sutures. In the end, the fascial defect and incision were sutured conventionally after the removal of stoma remnant.

For the conventional open ileostomy reversal, the skin, subcutis and fascia were successively dissected to recognize the proximal and distal ileum. Meanwhile, the tissue adhesion around the stoma was also separated using ultrasonic scalpels. The proximal and distal ileums around the stoma were exteriorized through the incision. Subsequently, a side-to-side anastomosis was extracorporeally conducted by linear staplers (Johnson ECR60B). Similar to TLAP group, the mesenteric defect was consolidated by absorbent threads and the abdominal cavity was closed.

### Data collection and variables definition

Gender, age, body mass index (BMI), American Society of Anesthesiologists (ASA) score, duration of ileostomy, history of preoperative adjuvant therapy before reversal, indication for antecedent operation and comorbidities were collected as baseline characteristics of patients. Operative time was calculated from first skin incision was made to final skin closure. Estimated blood loss was the sum of the blood volume collected within the suction canister after subtracting the amount of irrigation fluid used and the increased weight of used sponges (1 ml of blood is about 1g) based on previous studies (16, 17). The time of first ground activities and flatus passage was reported by patients. Postoperative hospitalization was defined as the number of nights from surgery to discharge. Short-term outcomes were defined as surgical results and postoperative complications within a follow-up period of 30-days after ileostomy reversal (18, 19).

### Statistical analysis

Demographic data, operation time, estimated blood loss, length of incision for stoma removal, first ground activities, first flatus passage, postoperative hospitalization days and complications were retrospectively analyzed. Here, the SPSS software of version 26.0 (SPSS Inc., Chicago, IL, USA) was used for statistical analysis. Quantitative data accorded with normal distribution checked by Shapiro-Wilk test were presented with mean ± standard derivation (SD) and compared with Student *t* test. In contrast, the skew distributional data were presented as median and interquartile range (IQR) and the Mann–Whitney U test was used to show the difference between TLAP and Open group. For categorical variables, data were presented with number and percentages and the *χ*^2^ test or Fisher's exact test was applied. Moreover, univariable logistic regression analysis was performed to estimate the odds of postoperative complication for each covariable. Subsequent multivariable logistic regression analysis was also constructed to adjust for the potential confounding effects of covariables. A *P*-value < 0.05 was considered as significant difference for all tests.

## Results

### General data

From September 2016 to September 2021, a total of 129 consecutive patients received loop ileostomy reversal were screened, of which 107 cases were found eligible and therefore included for subsequent analyses. Instead, 22 patients were excluded according to the exclusion criteria. The demographic and clinical parameters of all patients in this research are summarized in Table 1. In general, 48 patients received the TLAP technique while 59 cases received open surgery. With regard to baseline data, no difference was found between the TLAP and Open group on gender (*P* = 0.663), age (*P* = 0.568), BMI (*P* = 0.976), ASA score (*P* = 1), duration of ileostomy (*P* = 0.855) or preoperative neoadjuvant therapy (*P* = 0.520). Furthermore, 45.83% patients in TLAP group and 37.29% patients in Open group were with comorbidities even though the difference was not significant (*P* = 0.372). The most common comorbidity in TLAP group was hypertension, which accounted for 29.17% of the 48 patients. In contrast, diabetes mellitus was the most common comorbidity in Open group (*n* = 9, 15.25%). Other comorbidities included hyperthyroidism, coronary disease, renal insufficiency and multisystem diseases. In regards to the indication for antecedent operation, we found a significant difference according to *χ*^2^ test (*P* = 0.012) due to more patients with colon cancer received open loop ileostomy reversal (*n* = 18, 30.51%). Further univariable and multivariable analyses were applied to minimize the disequilibrium between two groups.

**Table 1:** Demographic and baseline characteristics of patients.

	TLAP group (*n* = 48)	Open group (*n* = 59)	*P-*value
Gender, *n* (%)			0.663
Male	24 (50)	32 (54.24)	
Female	24 (50)	27 (45.76)	
Age, years, median (IQR)	63 (55–66)	58 (52–66)	0.568
BMI, kg/m^2^, mean ± SD	24.181 ± 2.455	24.198 ± 3.110	0.976
ASA score, *n* (%)			1
1–2	45 (93.75)	56 (94.92)	
3–4	3 (6.25)	3 (5.09)	
Duration of ileostomy, mouths, median (IQR)	8.5 (6.25–12)	9 (7–14)	0.855
Preoperative neoadjuvant therapy, *n* (%)	28 (58.33)	38 (64.41)	0.520
Indication for antecedent operation, *n* (%)			0.012
Colon cancer	5 (10.42)	18 (30.51)	
Rectal cancer	43 (89.58)	41 (69.49)	
Comorbidities, *n* (%)	22 (45.83)	22 (37.29)	0.372
Hypertension	14 (29.17)	7 (11.86)	
Diabetes mellitus	2 (4.17)	9 (15.25)	
Hyperthyroidism	0	1 (1.69)	
Coronary disease	1 (2.08)	2 (3.39)	
Hypertension and diabetes mellitus	3 (6.25)	2 (3.39)	
Hypertension, coronary disease and renal insufficiency	2 (4.17)	1 (1.69)	

### Surgical parameters

In our institution, all enrolled patients received an equivalent perioperative management, including routine preoperative bowel preparation, prophylactic antibiotics application and enhanced recovery after surgery and postoperative analgesia. Surgical and postoperative indicators were presented in Table 2. Predictably, the median operation time of TLAP was 87.5 min, which was slightly longer than Open group (85 min) but with no statistical difference (*P* = 0.205). The estimated blood loss was also similar (*P* = 0.622). However, enough incisive length appeared to be inessential for TLAP group, resulting in a reduced incision length compared to Open group (4.5 cm vs. 6 cm, *P* < 0.001).

**Table 2:** Surgical and postoperative parameters of patients.

	TLAP group (*n* = 48)	Open group (*n* = 59)	*P-*value
Operation time, min, median (IQR)	87.5 (70–107.75)	85 (68–100)	0.205
Estimated blood loss, ml, median (IQR)	20 (10–30)	20 (10–30)	0.622
Length of incision, cm, median (IQR)	4.5 (3–6)	6 (5–8)	<0.001
First ground activities, days, median (IQR)	1 (1–1)	2 (1–2)	<0.001
First flatus passage, days, median (IQR)	2 (2–3)	3 (2–3)	0.004
Postoperative hospitalization, days, median (IQR)	5 (3–6.75)	6 (5–7)	0.007
Postoperative complications	3	10	0.026
Incisional infection	3	6	
Intra-abdominal infection	0	1	
Incision fat liquefaction	0	1	
Intestinal obstruction	0	1	
Anastomotic bleeding	0	1	

### Postoperative recovery and complications

Of note, the median time of first ground activities in TLAP patients was the 1st day, exhibiting a faster recovery compared to the Open group (2nd day, *P* < 0.001). Besides, a quicker flatus passage (2 days vs. 3 days, *P* = 0.004) and shorter postoperative hospitalization day (5 days vs. 6 days, *P* = 0.007) were also found in TLAP group. More importantly, we found a significant difference of postoperative complications between TLAP and Open group (3 vs. 10, *P* = 0.026). Specifically, the most common complication was incisional infection (*n* = 8), which was more prevalent among the Open group (*n* = 6). Some characteristic complications were found in Open group, including intra-abdominal infection (*n* = 1), incision fat liquefaction (*n* = 1), intestinal obstruction (*n* = 1) and anastomotic bleeding (*n* = 1). These results indicated a reduced incidence of postoperative complication after TLAP reversal.

To maximumly eliminate the difference of baseline characteristics and explore factors associated with post-operation complications, logistic regression analyses were performed (Table 3). From the univariable analyses, patients conducted conventional open reversal had over 3-fold increase in odds of complication compared with patients received TLAP (OR = 3.073; CI , 1.109–8.516; *P* = 0.031). Univariable analysis also showed that higher BMI index was associated with significantly increased risk of postsurgical complication (OR = 0.834; CI, 0.704–0.986; *P* = 0.037). However, gender, age, ASA score, duration of ileostomy, neoadjuvant therapy history, antecedent tumor location and comorbidities were not associated with postoperative complications. Multivariable analysis also showed that surgical approaches (OR = 3.316; CI, 1.118–9.835; *P* = 0.031) and BMI (OR = 0.823; CI, 0.682–0.993; *P* = 0.042) were the only factor independently associated with postoperative complications when adjusted for the potentially confounding effects of baseline variables.

**Table 3:** Logistic regression analysis of potential factors related to postoperative complications.

	Univariable analysis		Multivariable analysis	
	Odds ratio (95% CI)	*P-*value	Odds ratio (95% CI)	*P-*value
Operation approach	3.073 (1.109–8.516)	0.031	3.316 (1.118–9.835)	0.031
Gender	0.575 (0.229–1.441)	0.238		
Age	1.022 (0.984–1.062)	0.257		
BMI	0.834 (0.704–0.989)	0.037	0.823 (0.682–0.993)	0.042
ASA	3.308 (0.320–34.224)	0.316		
Duration of ileostomy	0.998 (0.930–1.071)	0.964		
Preoperative neoadjuvant therapy	1.685 (0.631–4.503)	0.298		
Indication for antecedent operation	1.507 (0.307–7.399)	0.614		
Comorbidities	1.029 (0.409–2.588)	0.951		

## Discussion

With the improvement of surgical techniques and apparatuses, laparoscopic reversal has gained increasing attention. However, the advantage of laparoscopic reversal compared to the open style has not been fully explored. In this study, we retrospectively analyzed 107 colorectal cancer patients received either TLAP or open ileostomy reversal at our hospital. For the first time, we found that patients with TLAP reversal had smaller incisions, earlier off-bed ambulation and anal aerofluxus time as well as shorter hospitalization stay compared to the traditional open approach. More importantly, TLAP technique significantly reduced the incidence of postoperative complications based on logistic regression analyses. Taken together, these results suggested the satisfactory short-term outcomes of TLAP reversal and indicated its potential on reducing postoperative complications.

Consistent with previous findings (20), open ileostomy reversal resulted a 16.95% morbidity in our series. Moreover, patients received open reversal had 3-fold increase in odds of complication compared to those with TLAP (6.25%). These results suggested TLAP technique resulted a prominent reduction of postoperative complications. One possible explanation was partially attributed to the application of laparoscopy. In a randomized clinical trial (7), patients received laparoscopic separation of abdominal adhesions had a lower incidence of morbidity than open ileostomy reversal. Similar outcomes were also reported when evaluating postoperative complications between laparoscopic colostomy reversal and open procedures (21). As a result, these consistent results indicated that laparoscopic-assisted reversal could be advantageous in reducing complications. Notably, extracorporeal digestive reconstruction, which means the intestinal stump should be pulled out for anastomosis, might increase potential risks of intestinal obstruction and abdominal infection. In our study, the intestinal obstruction and intra-abdominal infection were reported only after open reversal. In contrast, the TLAP technique could reduce bowel manipulation, mesentery traction as well as incision length (22), which might provide another reasonable explanation of reduced postoperative complications.

Patients with TLAP showed quicker postoperative recovery than those with open reversal. In fact, accumulative studies have already demonstrated the strength of laparoscopic reversal on postoperative recovery. In 2021, Yellinek and his colleagues (23) found that laparoscopic closure significantly reduced the length of hospitalization, providing the superiority of laparoscopic technique. As one of the most challenging procedures in colorectal surgery, Hartmann reversal could also be performed in a laparoscopic way, significantly decreased hospital stay compared to traditional reversal (24). Furthermore, the technique of intracorporeal anastomosis could also contribute to postoperative recovery. Patients underwent TLAP right/left/transverse colectomy have exhibited faster recovery of gastrointestinal function than those with extracorporeal anastomosis (13, 25, 26). As a consequence, our results provided valuable evidence and exploration of intracorporeal anastomosis in ileostomy reversal.

Unexpectedly, we found a higher BMI could increase the incidence of postoperative complications. According to literature, the surgical site infection rate could be significantly increased in obese patients rather than those with normal BMI (27). Higher BMI index was also regarded as a predictive factor for conversion (28). These results are comprehensible due to the thickness of abdominal wall in obese patients. Interestingly, our previous study focusing on obese patients showed TLAP reversal could significantly reduce the incidence of incisional infection (10). As a result, this current research provided a complementary result, indicating the promising application of TLAP reversal for obesity patients. In contrast, inconsistent with previous report, we found the TLAP reversal took more time compared to the open group but the difference was not significant. A possible explanation is that although the TLAP technique theoretically enables easier surgery process, its strength might be embodied to the most extend on obesity patients.

Admittedly, this study was conducted retrospectively that the baseline data could not be fully balanced. However, univariable and multivariable analyses were used to maximumly diminish the potential influences. Although the cost analysis assessment between TLAP and open technique was not examined, TLAP might not significantly increase hospitalization expenses as accumulative studies showed the intracorporeal anastomosis would not lead to higher expenses (12).

There are some limitations that should be noted in this study. Firstly, since growing number of patients chose a more minimally invasive approach for ileostomy reversal, the sample size of open reversal after the second half of 2018 were small (*n* = 10), leading potential selection bias and time-dependent confounding bias. Secondly, TLAP reversal required a learning process for master whereas the technique of open reversal could be considered proficient. As a result, surgical experience might have an impact on perioperative outcomes. Lastly, this study was only focused on the 30-day outcomes after reversal. Further large-sample and long-time follow-up should be performed.

In conclusion, this study compared the short-term outcomes between the TLAP and open reversal. We found the TLAP reversal could reduce wound length, promote gastrointestinal function recovery and decrease the incidence of postoperative complications. These results indicated the satisfactory short-term outcomes of TLAP, providing reliable evidence of its potential on colorectal cancer patients.

## Data Availability

The original contributions presented in the study are included in the article/Supplementary Material, further inquiries can be directed to the corresponding author/s.
